# Design and rationale of the AngioSeal versus the Radial approach In acute coronary SyndromE (ARISE) trial: a randomized comparison of a vascular closure device versus the radial approach to prevent vascular access site complications in non-ST-segment elevation acute coronary syndrome patients

**DOI:** 10.1186/1745-6215-14-435

**Published:** 2013-12-18

**Authors:** Pedro Beraldo de Andrade, Luiz Alberto Piva e Mattos, Marden André Tebet, Fábio Salerno Rinaldi, Vinícius Cardozo Esteves, Ederlon Ferreira Nogueira, João Ítalo Dias França, Mônica Vieira Athanazio de Andrade, Robson Alves Barbosa, André Labrunie, Alexandre Antônio Cunha Abizaid, Amanda Guerra de Moraes Rego Sousa

**Affiliations:** 1 Invasive Cardiology, Santa Casa de Marília, Avenida Vicente Ferreira, 828 - Cascata, Marília, São Paulo 17515-900, Brazil; 2 Hospital das Clínicas da Faculdade Estadual de Medicina de Marília, Marília, São Paulo Brazil; 3 Instituto Dante Pazzanese de Cardiologia, São Paulo, São Paulo Brazil; 4 Rede D’or de Hospitais do Brasil, São Paulo, São Paulo Brazil; 5 Hospital e Maternidade Brasil - Rede D’Or, Santo André, São Paulo Brazil; 6 Hospital do Coração de Londrina, Londrina, Paraná, Brazil

**Keywords:** Radial approach, Femoral approach, Vascular closure device, Angioplasty, Acute coronary syndrome, Access site complication

## Abstract

**Background:**

Arterial access is a major site of bleeding complications after invasive coronary procedures. Among strategies to decrease vascular complications, the radial approach is an established one. Vascular closure devices provide more comfort to patients and decrease hemostasis and need for bed rest. However, the inconsistency of data proving their safety limits their routine adoption as a strategy to prevent vascular complications, requiring evidence through adequately designed randomized trials. The aim of this study is to compare the radial versus femoral approach using a vascular closure device for the incidence of arterial puncture site vascular complications among non-ST-segment elevation acute coronary syndrome patients submitted to an early invasive strategy.

**Methods:**

ARISE is a national, multicenter, non-inferiority randomized clinical trial. Two hundred patients with non-ST-segment elevation acute coronary syndrome will be randomized to either radial or femoral access using a vascular closure device. The primary outcome is the occurrence of vascular complications at an arterial puncture site 30 days after the procedure, including major bleeding, retroperitoneal hematoma, compartment syndrome, hematoma ≥ 5 cm, pseudoaneurysm, arterio-venous fistula, infection, limb ischemia, arterial occlusion, adjacent nerve injury or the need for vascular surgical repair.

**Results:**

Enrollment was initiated in September 2012, and until October 2013 91 patients were included. The inclusion phase is expected to last until the second half of 2014.

**Conclusions:**

The ARISE trial will help define the role of a vascular closure device as a bleeding avoidance strategy in patients with NSTEACS.

**Trial registration:**

ClinicalTrials.gov identifier: NCT01653587

## Background

Antithrombotic therapy and percutaneous or surgical myocardial revascularization procedures represent the basis of hospital treatment for patients admitted with non-ST-segment elevation acute coronary syndrome (NSTEACS) [1,2]. However, the desired reduction of ischemic event recurrence is accompanied by an increased incidence of bleeding [3,4]. Initially tolerated as an inherent complication of anti-ischemic treatment, major bleeding is now recognized as a predictor of mortality and adverse ischemic outcomes [5]. In fact, NSTEACS patients who develop major bleeding have a two- to ten-fold increase in mortality after a one-year follow-up [6,7]. In the Acute Catheterization and Urgent Intervention Triage Strategy (ACUITY) trial [8] involving 13,819 NSTEACS patients submitted to an early invasive strategy and randomized for antithrombotic treatment with unfractionated heparin (UFH) plus IIb-IIIa glycoprotein receptor inhibitor (GPI), bivalirudin plus GPI or bivalirudin alone, the prognostic impact of major bleeding was comparable to that of acute myocardial infarction (AMI) in subsequent mortality (11.7% versus 9.1%, respectively) [9]. Randomized clinical trials involving NSTEACS patients show major bleeding rates varying from 2% to 5% [5,8], approaching the prevalence of refractory ischemia, AMI or deaths observed in these studies. A publication of the National Cardiovascular Data Registry Acute Coronary Treatment and Intervention Outcomes Network Registry Get with the Guidelines (NCDR ACTION Registry-GWTG) [10], a representative registry of real world practice, has evaluated 72,699 unselected patients with NSTEACS and 48,943 patients with ST-segment-elevation AMI evaluated at 360 North American hospitals between January 2007 and June 2009. Authors have reported a major bleeding rate of approximately 9% among NSTEACS patients and 12% among those with ST-segment-elevation AMI, primarily influenced by the presence of comorbidities such as older age, female gender, chronic renal failure, as well as the use of invasive techniques [9,11].

### Bleeding and vascular complications related to arterial access

Because arterial puncture followed by sheath insertion using the modified Seldinger technique [12] has become the standard method used to perform invasive cardiovascular procedures, vascular access has become a major site of bleeding complications [13]. According to the Global Registry of Acute Coronary Events (GRACE), the most frequent bleeding sites were gastrointestinal (31.5%) and those related to vascular access (23.8%), with the latter being more prevalent among patients submitted to invasive strategies [11]. In a joint analysis of 17,393 acute coronary syndrome (ACS) patients submitted to percutaneous coronary intervention (PCI) and included in the studies Randomized Evaluation in PCI Linking Angiomax to Reduced Clinical Events (REPLACE) - 2, ACUITY and Harmonizing Outcomes with RevascularIZatiON and Stents in Acute Myocardial Infarction (HORIZONS-AMI), the bleeding prevalence by Thrombosis in Myocardial Infarction (TIMI) criteria was 5.3%, of which 2.1% (38.6%) were related to vascular access [14].

### Strategies to prevent bleeding and vascular complications

#### Radial technique

Among the strategies to decrease vascular complications after invasive coronary procedures, the radial approach is an established one [15,16]. Although it has been adopted by only a few centers, it provides more comfort to patients, allows early ambulation, decreases hospital stay and shows less vascular puncture site complications [17]. In a comparative randomized trial between radial and femoral techniques involving 7,021 ACS patients submitted to invasive techniques, both techniques were shown to be safe and effective for PCI, with similar incidences of death, AMI, stroke and major bleeding at 30 days (3.7% versus 4.0%; *P* = 0.50) [18]. However, the radial approach displayed significantly decreased vascular complications, including pseudoaneurysm, large hematomas, arterio-venous fistula and limb ischemia requiring surgical intervention (1.4% versus 3.7%; *P* < 0.0001).

Given the small number of training centers that use the radial technique, the uncertainties concerning the learning curve which would be associated with a higher failure rate and more radiological exposure, and the lack of large-scale multicenter studies that can reproduce the excellent results obtained by highly experienced centers, the femoral approach remains the most popular for invasive coronary procedures [19].

#### Percutaneous vascular closure devices

Although the real prognostic significance of preventing vascular access complications and minor bleedings has not yet been established, the occurrence of vascular access complications is associated with poor patient adherence to the antiplatelet therapy after hospital discharge and is a major and known predictor of ischemic complication recurrence [20]. Strategies to decrease femoral access vascular complications have been frequently evaluated and implemented, such as the use of smaller diameter endovascular devices, early arterial sheath removal, fluoroscopy or ultrasound-guided femoral puncture and the choice of antithrombotic agents with better safety profiles [8,21-23].

Since 1995, percutaneous femoral vascular closure devices (VCD) were introduced to decrease vascular complications, hemostasis and ambulation times of patients submitted to invasive procedures by femoral access. Although these devices have been rapidly incorporated into interventionist practice, they have shown conflicting results regarding their safety and efficacy, motivating a class III recommendation as a strategy to decrease vascular complications in a recent position taken by the American Heart Association [24].

Three meta-analyses that compared VCD and manual compression from 2004 have reported conflicting results regarding their safety. Koreny *et al*. [25] grouped data from 30 randomized trials involving 4,000 patients and showed a decrease of 17 minutes to obtain hemostasis with VCD, at the expense of a non-significant increase in hematomas, bleeding, arterio-venous fistulas and pseudoaneurysms. Nikolsky *et al*. [26] identified 30 randomized and observational studies in 37,066 patients where VCD were associated with increased vascular complications compared with manual compression, particularly using the VasoSeal (Datascope Corp., Montvale, NJ) device. Conversely, Vaitkus *et al*. [27] described in a meta-analysis involving 15 randomized trials and 5,084 patients a decrease in vascular complications favoring the new technology, particularly when using AngioSeal (St. Jude Medical, St. Paul, MN) and Perclose (Abbott Vascular, Redwood City, CA) devices, an observation that has been validated by results of large multicenter observational registries published subsequently [28,29].

In summary, different VCD, although not demonstrating a class effect, clearly provide more comfort to patients, decreasing hemostasis and bed rest time. However, the inconsistency of data proving their safety limits their routine adoption as a strategy to prevent vascular complications, requiring evidence through adequately designed randomized studies. The hypothesis of this investigation is that among NSTEACS patients submitted to an early invasive strategy and randomized to the femoral or radial approach, the percutaneous femoral vascular closure device would decrease the prevalence of vascular complications at the puncture site, thus fulfilling the non-inferiority criteria compared with radial access.

## Methods

### The ARISE study

#### Design

**Figure 1:**
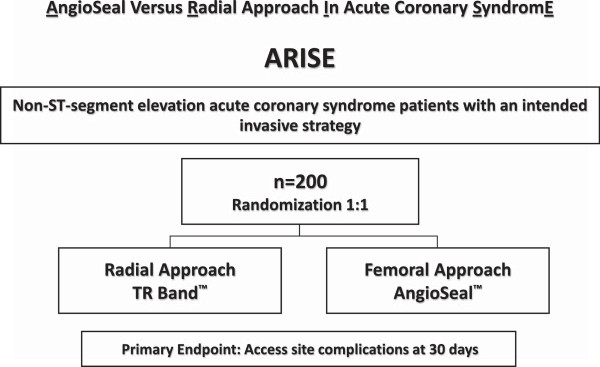
ARISE study design: a randomized trial of the radial versus femoral approach using AngioSeal in non-ST-segment elevation acute coronary syndrome (NSTEACS) patients.

ARISE is a national, multicenter, non-inferiority randomized clinical trial comparing the radial versus femoral approach using VCD to decrease vascular complications related to an arterial puncture site among NSTEACS patients submitted to an early invasive strategy (Figure 1). The primary outcome will be evaluated from randomization until 30 days after the invasive coronary procedure.

#### Outcomes

##### Primary outcome

Vascular complications at the arterial puncture site 30 days after the procedure (vascular complications at the arterial puncture site include major bleeding, retroperitoneal hematoma, compartment syndrome, hematoma ≥ 5 cm, pseudoaneurysm, arterio-venous fistula, infection, limb ischemia, arterial occlusion including asymptomatic occlusion, adjacent nerve injury or the need for vascular surgical repair).

##### Secondary outcomes

Individual components of the primary outcome, hematoma < 5 cm, major bleeding unrelated to the puncture site or myocardial revascularization surgery, device success and crossover rate; cardiovascular death, AMI or stroke at 12 months of evolution.

Study outcome definitions are listed in Table 1. A committee of clinicians will adjudicate all primary outcomes.

**Table 1:** Outcome definitions

**Term**	**Definition**
Major bleeding	Type 3 ((3a) bleeding with hemoglobin drop ≥ 3 and < 5 g/dL, or packed red cells transfusion; (3b) bleeding with hemoglobin drop ≥ 5 g/dL, heart tamponade, bleeding requiring surgical intervention or bleeding requiring intravenous inotropic drugs; (3c) intracranial hemorrhage; subcategories confirmed by autopsy, imaging examinations or lumbar puncture; intraocular bleeding with vision impairment) or type 5 bleeding ((5a) possibly fatal bleeding, (5b) definitive fatal bleeding), according to the Bleeding Academic Research Consortium definition [30].
Retroperitoneal hematoma	Defined as building up of blood in the retroperitoneal space, caused by common femoral artery puncture above the inguinal ligament, not obtaining an adequate hemostasis with consequent hematoma formation. It is clinically manifested by dorsal, loin or inguinal pain, hypotension and/or hematocrit drop. Its diagnosis has to be confirmed by abdominal ultrasound and/or CT.
Compartment syndrome	Defined as abnormal tissue pressure increase inside an osteo-fascial compartment (considering in this analysis the involvement of upper or lower limbs after arterial puncture), impairing nervous and muscular structure irrigation, characterized by paresthesia, continuous pain, hypoesthesia, edema and stiffening of the affected region, peaking with tissue necrosis and/or permanent functional injury if not adequately treated.
Hematoma	Defined as localized collection of extravascular blood adjacent to the vessel, located in the topography of the punctured artery used to perform the procedure.
Pseudoaneurysm	Defined as a neocavity delimited by tissues adjacent to the injured vessel, fed by continuous blood flow into and out, coming from the real lumen through a narrow neck connecting it to the inside of the cavity. Diagnosis is determined by the presence of a pulsating bulge close to the puncture hole and is confirmed by duplex scanning.
Arterio-venous fistula	Defined as an abnormal and acquired communication between the arterial and venous surface caused by the inadvertent puncture of a vein adjacent to the femoral or radial artery. Diagnosis is determined by the presence of a continuous murmur or thrill at the puncture site and is confirmed by duplex scanning.
Infection	Defined as the introduction and/or colonization of microorganisms in structures adjacent to the puncture site and/or blood flow, predisposed by difficult access, repeated punctures, long introducer stay, multiple catheters or a prolonged procedure. It is manifested by pain, hyperemia, local edema, adenopathy, fever and/or leukocytosis with a shift to the left in the blood count.
Limb ischemia	Defined as the presence of signs and/or symptoms such as local pain, paresthesia, paresis, skin pallor, cyanosis, lack of pulse, cold extremities and/or muscle tenderness, caused by acute or sub-acute arterial occlusion and confirmed by duplex scanning and/or arteriography.
Asymptomatic arterial occlusion	Defined by blockade of the arterial blood flow without manifestations of disturbance of the cell mechanism or insufficient tissue blood supply, not involving the terminal arterial segment (example: radial artery occlusion).
Adjacent nerve injury	Defined by the presence of sensory and/or motor disorders in the limb through which the invasive coronary procedure was performed, with persistence of signs and/or symptoms for ≥ 24 hours, as a consequence of direct injury by inadvertent nerve puncture, excessive compression or extrinsic compression by a hematoma and/or a pseudoaneurysm.
Vascular surgical repair	Defined by the presence of complications at the arterial puncture site requiring immediate or late (first 15 days) surgical intervention to prevent and/or minimize sequelae to the affected limb.

#### Patient population

Patients with NSTEACS will be managed with an invasive approach if the following pre-requisites are met: (i) palpable radial artery with normal Allen or oximetry tests, (ii) familiarity of the operator with the radial (≥ 100 transradial coronary procedures per year with a femoral crossover rate ≤ 4%) and femoral techniques using VCD (≥ 50 transfemoral coronary procedures per year with AngioSeal) and (iii) agreement of the operator to use the access route determined by the randomization process (Table 2).

**Table 2:** Eligibility criteria

	
Inclusion criteria	Non-ST-segment elevation acute coronary syndrome patients (ischemic symptoms suspicious of non-ST-segment elevation ACS (unstable angina or non-ST-segment elevation AMI) defined as clinical presentation compatible with a new manifestation or worsening of chest pain characteristic of ischemia at rest or at minimum effort, lasting more than 10 minutes, and at least one of the following items: (a) ECG changes compatible with new ischemia (ST-segment depression of at least 1 mm, transient ST-segment elevation, ST-segment elevation ≤ 1 mm or T wave inversion > 3 mm in at least two contiguous shunts); (b) cardiac enzymes (CK-MB or troponin T or I) above the upper normality range limit; (c) patients > 60 years of age without ECG or myocardial necrosis marker changes; however, with previous documentation of coronary atherosclerotic disease, confirmed by previous hospitalization due to AMI, previous percutaneous or surgical myocardial revascularization procedure, significant coronary atherosclerotic disease confirmed by coronary angiography or positive functional test for myocardial ischemia)
	Intention to submit patient to an early invasive strategy consisting of coronary angiography immediately followed by PCI, when applicable, in the first 72 hours after admission
	Patient informed about the nature of the study and agreeing with its general terms and having signed the informed consent, as approved by the Research Ethics Committee of the respective center
	Patient eligible for coronary angiography and both radial and femoral PCI with the following pre-requisites: (a) palpable radial artery with the Allen or normal oximetry tests, (b) familiarity of the operator with the radial and femoral techniques using AngioSeal, (c) agreement of the operator to use the access route determined by the randomization process
Exclusion criteria	Less than 18 years of age
	Pregnancy
	Chronic use of vitamin K antagonists, direct thrombin inhibitors or oral factor Xa antagonists
	Hypersensitivity to antiplatelet and/or anticoagulant drugs
	Active bleeding or high bleeding risk (severe liver failure, active peptic ulcer, creatinine clearance < 30 mL/min, platelets count < 100,000 mm^3^)
	Uncontrolled systemic hypertension
	Cardiogenic shock
	Previous myocardial revascularization surgery with ≥ 1 internal mammary or radial artery graft
	Documented chronic peripheral arterial insufficiency preventing the use of the femoral technique
	Severe concomitant disease with life expectancy below 12 months
	Participation in drug or device investigative clinical trials in the last 30 days
	Indication of elective percutaneous coronary intervention to be performed at a moment different from immediately after coronary angiography
	Medical, geographic or social conditions impairing participation in the study or inability to understand and sign the informed consent term

#### Procedures

Patients admitted with NSTEACS who are scheduled for early invasive stratification by coronary angiography followed by PCI, when applicable, will be evaluated in terms of feasibility of both radial and femoral access procedures. After the evaluation, with the patient meeting all inclusion criteria and in the absence of exclusion criteria, the patient may be included in the study after signing the free and informed consent term.

Patients will be randomized for the radial or femoral technique with VCD using a randomized sequence obtained by computer algorithms and maintained in individual, opaque and closed envelopes to conceal the allocation process.

Both radial and femoral coronary angiography will be performed using the Judkins technique and 6-French diameter sheaths and pre-molded catheters for selective catheterization of the left and right coronary arteries, with the possibility to change the diameter of the devices at the operator’s discretion. PCI will be indicated when a lesion presumably responsible for the adjacent clinical event is identified, with a stenosis diameter severity ≥ 70% and a high probability of angiographic success; PCI is ideally performed immediately after coronary angiography and left ventriculography. Patients with multiarterial coronary disease will be submitted to PCI after agreement among the clinical cardiologist, interventionist and heart surgeon. Procedures will be performed according to recommendations and provisions of current guidelines.

#### Arterial homeostasis

##### Radial approach

The TR Band device (Terumo Medical Corporation, Tokyo, JP) will be applied to obtain homeostasis according to a previously validated protocol [31,32]. Immediately after procedure completion, the sheath is initially pulled by approximately 2 cm. The device is applied to the patient with the green marker (located in the center of the larger balloon) positioned exactly at the puncture hole to aid in the location, visualization and control of possible bleeding. The balloon is inflated with an adequate syringe injecting 15 mL of air with simultaneous and total sheath removal, resulting in the absence of active bleeding. From the fourth hour and at each subsequent hour (fifth and sixth hours), 5 mL of air is slowly removed, maintaining the balloon connected to the syringe and controlling the plunger with the thumb. If bleeding occurs during any stage of device removal, the volume of air needed for homeostasis is again injected, repeating the process 60 minutes later. If device failure occurs, homeostasis will be obtained using a compressive dressing with a porous elastic adhesive bandage (Tensoplast, Smith & Nephew, London, UK).

##### Femoral approach

The AngioSeal VCD, comprising an absorbable collagen sponge and absorbable polymer anchor with polylactic and polyglycolic acid connected by an absorbable self-adjustable suture, will be used for hemostasis. The device seals the arteriotomy hole between its two major components, the anchor and collagen sponge. Hemostasis is primarily obtained through mechanical means that is supplemented by collagen platelet aggregation-inducing properties. The device will be released according to the manufacturer’s recommendations. First, an insertion introducer together with a femoral artery arteriotomy hole locator will be positioned using a 0.035-inch-guidewire. Once blood reflow by the distal edge of the set is confirmed, the guidewire and insertion introducer are removed, keeping the arteriotomy locator in position. Next, the repair and hemostasis device are inserted through the locator, exposing the anchor in the intraluminal space. The retreat of this second set places the anchor against the internal puncture hole. Maintaining the retreat, the collagen sponge is then released in the external puncture hole at the same time that the anchor is sustained, resulting in effective and safe hemostasis by preventing intra-arterial collagen release. Total set removal is achieved using an absorbable suture wrapped in a plastic tube to manually compact the collagen sponge against the arterial wall. After the appearance of an opaque mark, the suture is cut, the compacting tube exerting compression may be removed, and the remaining suture is cut close to the skin. If the device fails, homeostasis will be obtained by manual compression. Patients will be allowed to walk immediately after the radial procedure, and one hour after bed rest in the supine position after the femoral procedure with AngioSeal.

Table 3 illustrates possible antithrombotic treatment. After successful PCI, anticoagulant therapy will be withdrawn. Drugs such as angiotensin-converting enzyme inhibitors or angiotensin receptor blockers, beta blockers and statins will be prescribed according to current secondary prevention guidelines.

**Table 3:** Adjunct antithrombotic therapy

**Site**	**Emergency unit**	**Catheter lab**	**Coronary unit and/or ward**	**Home use duration**
**Drug**				
Aspirin	300 mg orally	No	100 mg per day orally	100 mg per day orally, indefinitely
Clopidogrel	600 mg orally	No	75 mg per day orally	75 mg per day orally, 12 months
Prasugrel	60 mg orally	No	10 mg per day orally	10 mg per day orally, 12 months
Ticagrelor	180 mg orally	No	90 mg twice daily orally	90 mg twice daily orally, 12 months
Enoxaparin	1 mg per kg SC	0.3 mg per kg IV if the last dose is 8 to 12 hours 0.5 to 0.75 mg per kg IV if the last dose is >12 hours	1 mg per kg SC twice daily	No
Fondaparinux	2.5 mg SC	85 UI per kg IV UFH or 60 UI per kg IV UFH if GPI is scheduled	2.5 mg/SC/day	No
Abciximab	No	Intravenous loading dose of 0.25 mg per kg	0.125 mcg per kg per min for 12 hours, IV, without using HNF	No
Tirofiban	No	Intravenous loading dose of 25 mcg per kg	0.15 mcg per kg per min for 12 to 18 hours, IV, without using HNF	No

GPI, IIb-IIIa glycoprotein receptor inhibitor; UFH; unfractionated heparin, IV, intravenous; SC subcutaneous.

Electrocardiogram (ECG), CK-MB and/or troponin, glucose, creatinine, sodium, potassium, blood count and coagulation tests shall be obtained before patient randomization. Biomarkers (CK-MB and/or troponin) shall be checked between 12 and 24 hours after the procedure. ECG shall be performed soon after the procedure and within 12 to 24 hours after the procedure or when a new ischemic event is suspected. Hemoglobin and hematocrit dosage will be required in the presence of vascular complications or bleeding. Vascular and systemic complications related to the arterial vascular access will be evaluated in the interval between procedure completion, hospital stay and the first 30 days of evolution, through a scheduled visit. Other outcomes will be considered until the 30 days of evolution, with late follow-up by a visit or telephone call regarding cardiovascular death, AMI or stroke, at 12 months.

#### Statistical analysis

The primary analysis of the study is a non-inferiority comparison between the radial approach and femoral approach using VCD to decrease vascular complications at the arterial puncture site, in all randomized patients, based on the intention-to-treat principle.

Estimating a vascular complication rate pre-specified in the primary outcome of approximately 3% for the radial technique [19,32] and 12% for the femoral technique [26-31] and determining a non-inferiority margin of 0.15 based on historical data [18,19] with an alpha level of 0.05 and a beta level of 0.10, the minimum estimated sample size per group was established as 97 individuals. Twenty additional patients may be added to the final population to correct any subsequent loss of follow-up. This non-inferiority margin was derived from a trial that demonstrate the benefit of radial approach on the reduction of major vascular complications (hazard ratio, 0.37; 95% confidence interval, 0.27 to 0.52) as compared with femoral approach [18]. The non-inferiority margin of 0.15 was chosen in order to avoid a loss of greater than half the lower bound of the 95% confidence interval (0.27). The non-inferiority of the femoral approach with VCD will be declared if the lower confidence interval limit of 95% of the difference of both techniques does not include the specified inferiority margin value.

Categorical data will be presented as frequencies and group percentage and will be compared by chi-squared or Fisher’s exact tests. Continuous variables will be expressed as means and standard deviation and will be compared by Student’s *t-*test. Statistical analyses will be performed using Statistical Package for the Social Sciences for Windows (version 16.0; SPSS, Chicago, IL, USA).

The study was approved by the Dante Pazzanese Institute of Cardiology ethical committee, predicting and validating the Santa Casa de Marília as a co-participating institution. No extramural funding will be used to support this work. The authors are solely responsible for the design and conduct of this study, all study analyses and drafting and editing of the paper.

## Results and discussion

### Study status

Enrollment was initiated in September 2012, and until October 2013 91 patients were included. The inclusion phase is expected to last until the second half of 2014.

## Conclusions

Despite the proven efficacy of the radial approach in reducing vascular complications at the puncture site, the femoral approach remains the preferred technique at many centers worldwide. The ARISE trial will help define the role of vascular closure devices as a bleeding avoidance strategy in patients with NSTEACS.

## Abbreviations

ACS: Acute coronary syndrome; AMI: Acute myocardial infarction; ECG: Electrocardiogram; GPI: IIb-IIIa glycoprotein receptor inhibitor; NSTEACS: Non-ST-segment elevation acute coronary syndrome; PCI: Percutaneous coronary intervention; TIMI: Thrombosis in myocardial infarction; UFH: Unfractionated heparin; VCD: Vascular closure devices.

## Competing interests

The authors declare that they have no competing interests related to this study.

## Authors' contributions

All authors read and approved the final manuscript. PA participated in the design of the study, draft of the manuscript and coronary invasive procedures performance. LM participated in the design of the study and draft of the manuscript. MT participated in coronary invasive procedures performance. FR participated in coronary invasive procedures performance. VE participated in the primary and secondary outcome events adjudication. EN participated in coronary invasive procedures performance. JI participated in statistical analysis. MA participated in the primary and secondary outcome events adjudication. RB participated in the primary and secondary outcome events adjudication. AL participated in the primary and secondary outcome events adjudication. AA participated in the design of the study. AS participated in the design of the study.
